# ^68^Ga-Labeled [Thz^14^]Bombesin(7–14) Analogs: Promising GRPR-Targeting Agonist PET Tracers with Low Pancreas Uptake

**DOI:** 10.3390/molecules28041977

**Published:** 2023-02-20

**Authors:** Lei Wang, Ivica Jerolim Bratanovic, Zhengxing Zhang, Hsiou-Ting Kuo, Helen Merkens, Jutta Zeisler, Chengcheng Zhang, Ruiyan Tan, François Bénard, Kuo-Shyan Lin

**Affiliations:** 1 Department of Molecular Oncology, BC Cancer, Vancouver, BC V5Z 1L3, Canada; 2 Department of Radiology, University of British Columbia, Vancouver, BC V5Z 1M9, Canada; 3 Department of Functional Imaging, BC Cancer, Vancouver, BC V5Z 4E6, Canada

**Keywords:** gastrin-releasing peptide receptor, agonist, positron emission tomography, Gallium-68, pancreas uptake

## Abstract

With overexpression in various cancers, the gastrin-releasing peptide receptor (GRPR) is a promising target for cancer imaging and therapy. However, the high pancreas uptake of reported GRPR-targeting radioligands limits their clinical application. Our goal was to develop ^68^Ga-labeled agonist tracers for detecting GRPR-expressing tumors with positron emission tomography (PET), and compare them with the clinically validated agonist PET tracer, [^68^Ga]Ga-AMBA. Ga-TacBOMB2, TacBOMB3, and TacBOMB4, derived from [Thz^14^]Bombesin(7–14), were confirmed to be GRPR agonists by a calcium mobilization study, and their binding affinities (K_i_(GRPR)) were determined to be 7.62 ± 0.19, 6.02 ± 0.59, and 590 ± 36.5 nM, respectively, via in vitro competition binding assays. [^68^Ga]Ga-TacBOMB2, [^68^Ga]Ga-TacBOMB3, and [^68^Ga]Ga-AMBA clearly visualized PC-3 tumor xenografts in a PET imaging study. [^68^Ga]Ga-TacBOMB2 showed comparable tumor uptake but superior tumor-to-background contrast ratios when compared to [^68^Ga]Ga-AMBA. Moreover, [^68^Ga]Ga-TacBOMB2 and [^68^Ga]Ga-TacBOMB3 showed a much lower rate of uptake in the pancreas (1.30 ± 0.14 and 2.41 ± 0.72%ID/g, respectively) than [^68^Ga]Ga-AMBA (62.4 ± 4.26%ID/g). In conclusion, replacing Met^14^ in the GRPR-targeting sequence with Thz^14^ retains high GRPR-binding affinity and agonist properties. With good tumor uptake and tumor-to-background uptake ratios, [^68^Ga]Ga-TacBOMB2 is promising for detecting GRPR-expressing tumors. The much lower pancreas uptake of [^68^Ga]Ga-TacBOMB2 and [^68^Ga]Ga-TacBOMB3 suggests that [Thz^14^]Bombesin(7–14) is a promising targeting vector for the design of GRPR-targeting radiopharmaceuticals, especially for radioligand therapy application.

## 1. Introduction

The gastrin-releasing peptide receptor (GRPR) is a transmembrane G protein-coupled receptor (GPCR) that is expressed in the central nervous system, gastrointestinal tract, and pancreas [1] and regulates a variety of physiological functions, such as synaptic plasticity, hormone secretion, smooth muscle contraction, and cell proliferation [1,2,3]. Furthermore, GRPR has been shown to be overexpressed in a variety of malignancies [4,5,6,7,8,9,10] and is involved in a large array of pathophysiological conditions, such as associations with some neurochemical alterations in neurological disorders, the development of malignant neoplasms, and the proliferation of cancer cells in several cancer types [1,3,11,12,13]. The overexpression of GRPR in malignant tissues makes it a promising target for the design of targeted radiopharmaceuticals for the diagnosis and radioligand therapy of GRPR-expressing cancer.

Gastrin-releasing peptides (GRPs) and bombesin (BBN) are two natural GRPR ligands. GRPs and BBN share the same heptapeptide sequence at the C-terminus, which has been used as the targeting vector for the design of GRPR-targeting radiopharmaceuticals for cancer diagnosis and radioligand therapy for decades [14,15,16,17,18,19,20,21]. Some of the reported GRPR-targeting radioligands have been evaluated in the clinic [15,16,17,18,19,20]. However, all clinically evaluated GRPR-targeting radioligands have shown an extremely high uptake in the pancreas [15,19,20,22]. The high pancreas uptake limits the application of these GRPR-targeting radiopharmaceuticals for detecting cancer lesions adjacent to or located in the pancreas and lowers the maximum tolerated dose for targeted radioligand therapy to minimize toxicity.

**Figure 1:**
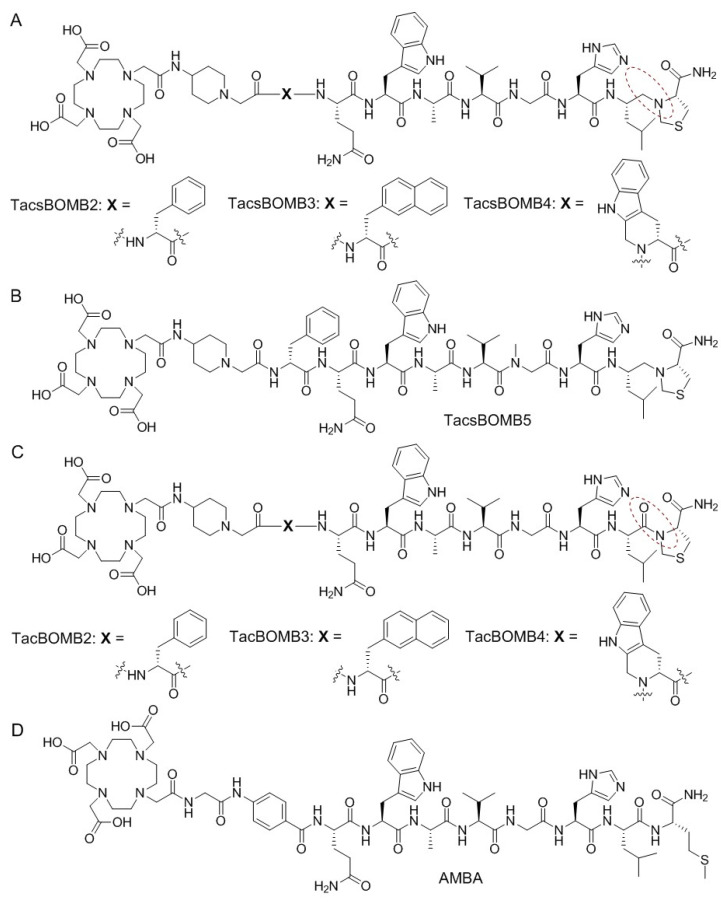
Chemical structures of (**A**) TacsBOMB2, TacsBOMB3, and TacsBOMB4; (**B**) TacsBOMB5; (**C**) TacBOMB2, TacBOMB3, and TacBOMB4; and (**D**) AMBA. The reduced peptide bond (inside the dashed brown circle) for the compounds in (**A**) is replaced with an amide bond (inside the dashed brown circle) for the compounds in (**C**).

The Schally group reported a series of GRPR-targeting ligands based on the bombesin(7–14) sequence by substituting Met^14^ with Thz^14^ (thiazoline-4-carboxylic acid) and introducing a reduced peptide bond (CH_2_-N) between residues 13–14 (Leu^13^ψThz^14^) [23,24]. These ligands were confirmed to be GRPR antagonists, and some were proven to have very promising binding affinities toward GRPR (K_i_ at pM scale) and the ability to inhibit cancer cell proliferation [25,26,27]. Inspired by their work, our group recently reported a series of ^68^Ga-labeled, DOTA-conjugated, GRPR-targeting radioligands derived from the reported [Leu^13^ψThz^14^]Bombesin(7–14), (Figure 1A,B) [28]. Compared with the clinically validated [^68^Ga]Ga-RM2, [^68^Ga]Ga-TacsBOMB2 showed comparable PC-3 tumor uptake and tumor-to-background contrast ratios, while [^68^Ga]Ga-TacsBOMB5 showed superior PC-3 tumor uptake and tumor-to-background contrast ratios. Most importantly, at 1 h post-injection, the pancreas uptake values of [^68^Ga]Ga-TacsBOMB2 (2.81 ± 0.78%ID/g) and [^68^Ga]Ga-TacsBOMB5 (1.98 ± 0.10%ID/g) were much lower than that of [^68^Ga]Ga-RM2 (41.9 ± 10.1%ID/g) [28].

The development of GRPR-targeting radiopharmaceuticals has been focused on the use of antagonist sequences as the targeting vector in the past decade, partly due to their higher in vivo stability [29], potentially higher tumor uptake due to more binding sites than those available for agonists [30], and/or less short term adverse effects [31,32]. However, agonists can be internalized upon binding to GRPR and potentially lead to longer tumor retention [1,32,33], which might be preferable, especially for use in the development of radiotherapeutic agents. We hypothesized that (1) replacing the reduced peptide bond (Leu^13^ψThz^14^) in our previously reported Ga-TacsBOMB2, Ga-TacsBOMB3, and Ga-TacsBOMB4 (Figure 1A) with an amide bond would restore their GRPR agonist characterizations, and (2) the resulting ^68^Ga-labeled [Thz^14^]Bombesin(7–14) derivatives might retain the low pancreas uptake characteristics observed from their Leu^13^ψThz^14^ analogs.

Thus, in this study, we synthesized [Thz^14^]Bombesin(7–14)-derived TacBOMB2, TacBOMB3, and TacBOMB4 (Figure 1C, by replacing the reduced peptide bond (CH_2_-N) between residues 13–14 (Leu^13^ψThz^14^) with a normal amide bond. Their agonist properties were determined using an in vitro fluorescence-based calcium release assay. Their potential for imaging GRPR expression was evaluated through in vitro competition binding, positron emission tomography (PET) imaging, and ex vivo biodistribution studies in a preclinical prostate cancer model, and compared with a clinically validated GRPR agonist tracer, [^68^Ga]Ga-AMBA (Figure 1D).

## 2. Results

### 2.1. Chemistry and Radiochemistry

DOTA-conjugated TacBOMB2, TacBOMB3, and TacBOMB4 were obtained in 30–55% yields, and their nonradioactive Ga-complexed standards were obtained in 58–82% yields. The HPLC conditions for their purification and MS characterizations are provided in Tables S1 and S2. Gallium-68 labeling was conducted in HEPES buffer (2 M, pH 5.0). After HPLC purification, ^68^Ga-labeled TacBOMB2, TacBOMB3, and AMBA were obtained in 51–80% decay-corrected radiochemical yields with 234–322 GBq/µmol molar activity and >95% radiochemical purity. The HPLC conditions for their purification and quality control are provided in Table S3.

### 2.2. Agonist Characterization, Binding Affinity, and Hydrophilicity

**Figure 2:**
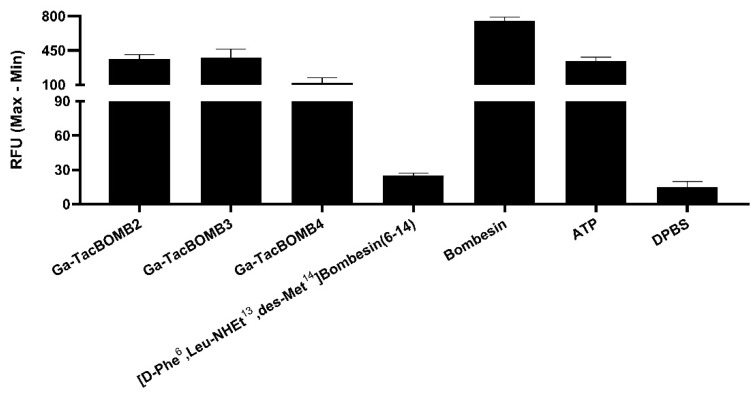
Intracellular calcium efflux in PC-3 cells induced by various tested ligands. Error bars indicate standard deviation.

Ga-TacBOMB2, Ga-TacBOMB3, and Ga-TacBOMB4 were confirmed to be GRPR agonists via intracellular calcium release assays using PC-3 cells (Figure 2). ATP (50 nM), as a positive control, and bombesin (50 nM), as an agonist control, induced Ca^2+^ efflux corresponding to 334 ± 39.0 and 754 ± 38.3 relative fluorescence units (RFUs), respectively. For 50 nM of Ga-TacBOMB2, Ga-TacBOMB3, and Ga-TacBOMB4, 361 ± 46.8, 378 ± 87.8, and 121 ± 52.3 RFUs were observed, respectively, which were significantly higher than the 14.9 ± 4.93 and 25.3 ± 1.92 RFUs recorded from the blank control (Dulbecco’s phosphate-buffered saline, DPBS) and the antagonist control ([D-Phe^6^,Leu-NHEt^13^,des-Met^14^]Bombesin(6–14), 50 nM), respectively.

**Figure 3:**
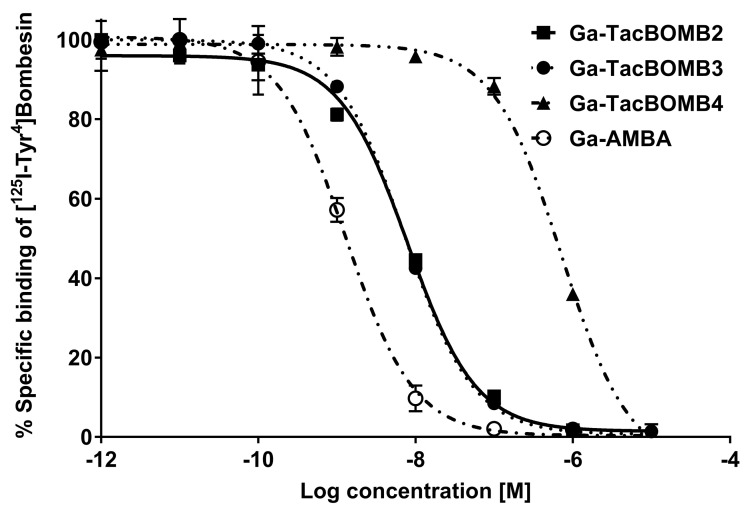
Displacement curves of [^125^I-Tyr^4^]Bombesin by Ga-TacBOMB2, Ga-TacBOMB3, Ga-TacBOMB4, and Ga-AMBA generated using GRPR-expressing PC-3 cells. Error bars indicate standard deviation.

Ga-TacBOMB2, Ga-TacBOMB3, Ga-TacBOMB4, and Ga-AMBA inhibited the binding of [^125^I-Tyr^4^]Bombesin to PC-3 cells in a dose-dependent manner (Figure 3). The calculated K_i_ values for Ga-TacBOMB2, Ga-TacBOMB3, Ga-TacBOMB4, and Ga-AMBA were 7.62 ± 0.19, 6.02 ± 0.59, 590 ± 36.5, and 0.99 ± 0.08 nM, respectively (*n* = 3). The hydrophilicity of [^68^Ga]Ga-TacBOMB2, [^68^Ga]Ga-TacBOMB3, and [^68^Ga]Ga-AMBA were measured using the shake flask method, and their LogD_7.4_ values were calculated to be −3.21 ± 0.03, −2.55 ± 0.03, and −3.66 ± 0.29, respectively (*n* = 3).

### 2.3. PET Imaging and Ex Vivo Biodistribution

**Figure 4:**
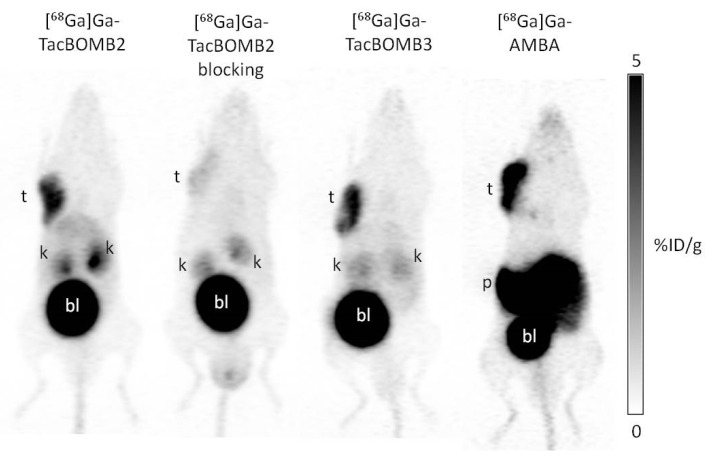
Representative PET images of [^68^Ga]Ga-TacBOMB2, [^68^Ga]Ga-TacBOMB3, and [^68^Ga]Ga-AMBA acquired at 1 h post-injection in mice bearing PC-3 tumor xenografts. t: tumor; k: kidney; p/i: pancreas/intestines; bl: urinary bladder.

The PC-3 tumor xenografts were clearly visualized in PET images acquired at 1 h post-injection using [^68^Ga]Ga-TacBOMB2, [^68^Ga]Ga-TacBOMB3, and [^68^Ga]Ga-AMBA (Figure 4). Both [^68^Ga]Ga-TacBOMB2 and [^68^Ga]Ga-TacBOMB3 were primarily excreted via the renal pathway, with low uptake in most background organs/tissues. [^68^Ga]Ga-TacBOMB2 had a better tumor-to-background contrast than [^68^Ga]Ga-TacBOMB3 and [^68^Ga]Ga-AMBA. [^68^Ga]Ga-AMBA showed a very high pancreas uptake, while the pancreases were invisible in the PET images of [^68^Ga]Ga-TacBOMB2 and [^68^Ga]Ga-TacBOMB3. Co-injection with 100 μg of [D-Phe^6^,Leu-NHEt^13^,des-Met^14^]Bombesin(6–14) significantly decreased the uptake of [^68^Ga]Ga-TacBOMB2 in PC-3 tumor xenografts.

**Figure 5:**
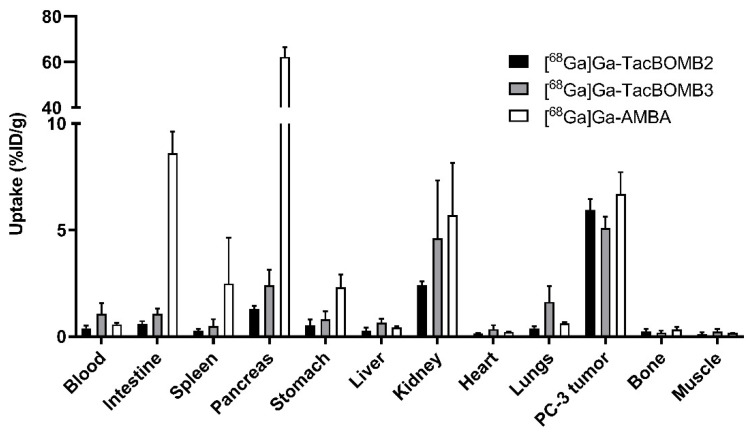
Uptake of [^68^Ga]Ga-TacBOMB2, [^68^Ga]Ga-TacBOMB3, and [^68^Ga]Ga-AMBA in PC-3 tumor xenografts and major organs/tissues of mice at 1 h post-injection. Error bars indicate standard deviation (*n* = 4).

**Figure 6:**
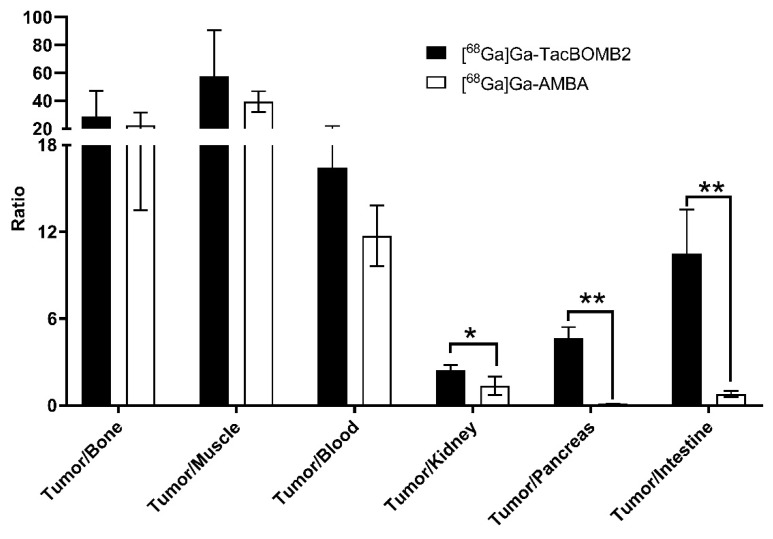
Comparison of tumor-to-organ contrast ratios of [^68^Ga]Ga-TacBOMB2 and [^68^Ga]Ga-AMBA obtained from PC-3 tumor-bearing mice at 1 h post-injection. Error bars indicate standard deviation (*n* = 4). * *p* < 0.05; ** *p* < 0.01.

**Figure 7:**
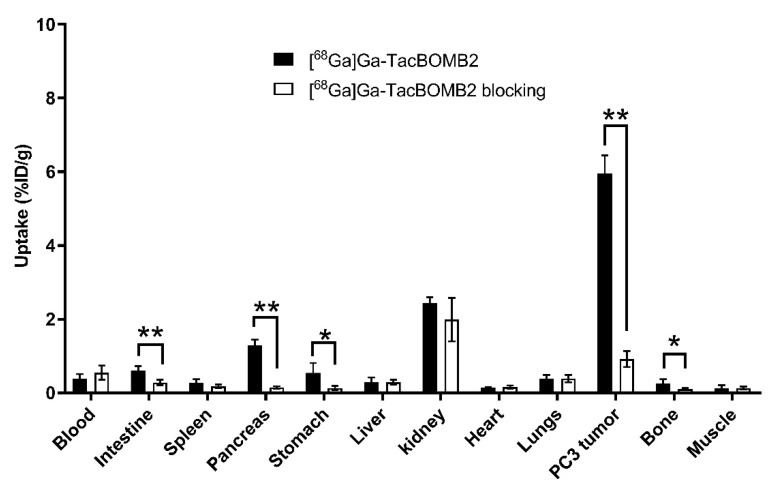
Comparison of [^68^Ga]Ga-TacBOMB2 with/without co-injection of [D-Phe^6^,Leu-NHEt^13^,des-Met^14^]Bombesin(6–14) on the uptake in PC-3 tumor xenografts and major organs/tissues in mice at 1 h post-injection. Error bars indicate standard deviation (*n* = 4). * *p* < 0.05; ** *p* < 0.01.

Biodistribution studies were performed at 1 h post-injection with ^68^Ga-labeled TacBOMB2, TacBOMB3, and AMBA in PC-3 tumor-bearing mice. Biodistribution results are consistent with the observations from their PET images and are provided in Figure 5, Figure 6 and Figure 7 and Table S4. PC-3 tumor uptake values for [^68^Ga]Ga-TacBOMB2, [^68^Ga]Ga-TacBOMB3, and [^68^Ga]Ga-AMBA were 5.95 ± 0.50, 5.09 ± 0.54, and 6.69 ± 1.03%ID/g, respectively. Pancreas uptake values for [^68^Ga]Ga-TacBOMB2, [^68^Ga]Ga-TacBOMB3, and [^68^Ga]Ga-AMBA were 1.30 ± 0.14, 2.41 ± 0.72, and 62.4 ± 4.26%ID/g, respectively. Intestine uptake values for [^68^Ga]Ga-TacBOMB2, [^68^Ga]Ga-TacBOMB3, and [^68^Ga]Ga-AMBA were 0.60 ± 0.12, 1.06 ± 0.26, and 8.62 ± 4.26%ID/g, respectively. Uptake values for the brain, muscle, fat, bone, liver, stomach, heart, and spleen were <1%ID/g for both [^68^Ga]Ga-TacBOMB2 and [^68^Ga]Ga-TacBOMB3.

Although the tumor uptake values of [^68^Ga]Ga-AMBA and [^68^Ga]Ga-TacBOMB2 were comparable, [^68^Ga]Ga-TacBOMB2 showed better tumor-to-organ uptake ratios in some major organs/tissues, such as bone, muscle, blood, kidney, pancreas, and intestine (Figure 6 and Table S4). The tumor-to-pancreas uptake ratio of [^68^Ga]Ga-AMBA (0.11 ± 0.01) was much lower than that of [^68^Ga]Ga-TacBOMB2 (4.64 ± 0.77, *p* < 0.01). Similarly, the tumor-to-intestine uptake ratio of [^68^Ga]Ga-AMBA was 0.79 ± 0.22, which was also much lower than that of [^68^Ga]Ga-TacBOMB2 (10.5 ± 3.06, *p* < 0.01).

The co-injection of [D-Phe^6^,Leu-NHEt^13^,des-Met^14^]Bombesin(6–14) reduced the uptake of [^68^Ga]Ga-TacBOMB2 in PC-3 tumor xenografts by 85% (from 5.95 ± 0.50 to 0.92 ± 0.22%ID/g, *p* < 0.01) at 1 h post-injection. Similarly, a significant reduction in the average uptake of [^68^Ga]Ga-TacBOMB2 was also observed in the intestines, pancreas, and stomach (Figure 7).

### 2.4. In Vivo Stability

[^68^Ga]Ga-TacBOMB2, [^68^Ga]Ga-TacBOMB3, and [^68^Ga]Ga-AMBA were shown to have limited in vivo stability in NRG mice (*n* = 3) (Figures S1–S3). Only 12.7 ± 2.93% of [^68^Ga]Ga-TacBOMB2 was found intact in plasma at 15 min post-injection, which was significantly lower than the intact fraction of [^68^Ga]Ga-AMBA (39.4 ± 10.8%, *p* = 0.01). The difference between the intact fraction of [^68^Ga]Ga-TacBOMB3 (27.3 ± 4.84%) and [^68^Ga]Ga-AMBA was not statistically significant (*p* = 0.15). Conversely, no intact [^68^Ga]Ga-TacBOMB2, [^68^Ga]Ga-TacBOMB3, or [^68^Ga]Ga-AMBA was detected in the mouse urine samples collected at 15 min post-injection (Figures S1–S3).

## 3. Discussion

The Schally group published a series of GRPR antagonists with picomolar binding affinity, including RC-3950-II (D-Phe-[Leu^13^ψThz^14^]Bombesin(7–14)), RC-3965-II (D-2-Nal-[Leu^13^ψThz^14^]Bombesin(7–14)), and RC-3910-II ((D-Tpi-[Leu^13^ψThz^14^]Bombesin(7–14))) [23,24]. Based on these three peptides, our group developed three Ga-complexed, DOTA-conjugated, GRPR-targeting ligands, Ga-TacsBOMB2, Ga-TacsBOMB3, and Ga-TacsBOMB4 (Figure 1A), respectively [28]. All Ga-TacsBOMB2, Ga-TacsBOMB3, and Ga-TacsBOMB4 ligands were also confirmed to be GRPR antagonists. In this study, we replaced their C-terminal reduced peptide bond (Leu^13^ψThz^14^) with a normal amide bond and investigated whether the resulting Ga-TacBOMB2, Ga-TacBOMB3, and Ga-TacBOMB4 (Figure 1C) restored agonist characteristics as well as their potential for PET imaging.

Intracellular calcium release assays revealed that all Ga-TacBOMB2, Ga-TacBOMB3, and Ga-TacBOMB4 ligands induced significantly more intracellular Ca^2+^ efflux compared to the antagonist control, [D-Phe^6^,Leu-NHEt^13^,des-Met^14^]Bombesin(6–14) and the blank control, DPBS (Figure 2). This observation confirmed the agonist property of Ga-TacBOMB2, Ga-TacBOMB3, and Ga-TacBOMB4, which are different from those previously reported GRPR antagonists, Ga-TacsBOMB2, Ga-TacsBOMB3, and Ga-TacsBOMB4 [28]. This confirms that replacing the C-terminal-reduced peptide bond (Leu^13^ψThz^14^) of Ga-TacsBOMB2, Ga-TacsBOMB3, and Ga-TacsBOMB4 with a normal amide bond restores their agonist characteristics. As the internalization of GRPR agonists after receptor binding may potentially result in a longer tumor retention period [1,32,33], radiolabeled [Thz^14^]Bombesin(7–14)-derived, GRPR-targeting ligands may be preferable to [Leu^13^ψThz^14^]Bombesin(7–14) derivatives, especially for radioligand therapy applications.

We further determined the binding affinities of these three GRPR-targeting ligands by conducting an in vitro competition binding assay. The K_i_ values of Ga-TacBOMB2 (7.62 ± 0.19 nM) and Ga-TacBOMB3 (6.02 ± 0.59 nM) were comparable, while Ga-TacBOMB4 showed a much poorer binding affinity to GRPR (K_i_ = 590 ± 36.5 nM). This finding is consistent with our previous report that replacing D-Phe in Ga-TacsBOMB2 with D-2-Nal doesn’t affect the binding affinity towards GRPR, while replacing D-Phe with D-Tpi leads to a significantly lower binding to GRPR. One possible explanation is that the free rotation of the Pip linker and the Ga-DOTA complex is compromised by the rigidity of the secondary amino group of D-Tpi, which results in a significant loss of binding affinity to GRPR.

As Ga-TacBOMB4 showed inferior binding affinity to GRPR, we labeled only TacBOMB2 and TacBOMB3 with ^68^Ga for further in vivo evaluation. The hydrophilicity of [^68^Ga]Ga-TacBOMB2 and [^68^Ga]Ga-TacBOMB3 was estimated by measuring their LogD_7.4_ values and was then compared with that of [^68^Ga]Ga-AMBA. [^68^Ga]Ga-AMBA was confirmed to be the most hydrophilic tracer (LogD_7.4_ = −3.66 ± 0.29), followed by [^68^Ga]Ga-TacBOMB2 and [^68^Ga]Ga-TacBOMB3, with LogD_7.4_ values of −3.21 ± 0.03 and −2.55 ± 0.03, respectively. Based on our previous study, we found that replacing D-Phe in [^68^Ga]Ga-TacsBOMB2 with a bulkier D-2-Nal reduced the hydrophilicity (the average LogD_7.4_ value increased by 0.64 from −2.39 ± 0.13 for [^68^Ga]Ga-TacsBOMB2 to −1.75 ± 0.04 for [^68^Ga]Ga-TacsBOMB3) [28]. The reduction in hydrophilicity was also observed in this study by replacing D-Phe in [^68^Ga]Ga-TacBOMB2 with a bulkier D-2-Nal. The average LogD_7.4_ value increased by 0.66 from −3.21 ± 0.03 for [^68^Ga]Ga-TacBOMB2 to −2.55 ± 0.03 for [^68^Ga]Ga-TacBOMB3, which is consistent with the 0.64 increase in the previous report, by converting [^68^Ga]Ga-TacsBOMB2 to [^68^Ga]Ga-TacsBOMB3. Similarly, we also observed that the amide bond derivatives ([^68^Ga]Ga-TacBOMB2 and [^68^Ga]Ga-TacBOMB3) were more hydrophilic than their corresponding reduced-peptide-bond derivatives ([^68^Ga]Ga-TacsBOMB2 and [^68^Ga]Ga-TacsBOMB3). The LogD_7.4_ value reduced by ~0.80 when replacing a reduced peptide bond with an amide bond (a 0.82 reduction, from −2.39 ± 0.13 for [^68^Ga]Ga-TacsBOMB2 to −3.21 ± 0.03 for [^68^Ga]Ga-TacBOMB2; a 0.80 reduction, from −1.75 ± 0.04 for [^68^Ga]Ga-TacsBOMB3 to −2.55 ± 0.03 for [^68^Ga]Ga-TacBOMB3).

Both [^68^Ga]Ga-TacBOMB2 and [^68^Ga]Ga-TacBOMB3 clearly visualized the PC-3 tumor xenografts in their PET images, which confirms the good targeting capabilities of both tracers to GRPR-expressing tumors (Figure 4). The biodistribution results of [^68^Ga]Ga-TacBOMB2 and [^68^Ga]Ga-TacBOMB3 were consistent with the observations from their PET images. Both [^68^Ga]Ga-TacBOMB2 and [^68^Ga]Ga-TacBOMB3 showed good uptake in PC-3 tumor xenografts, with uptake values of 5.95 ± 0.50 and 5.09 ± 0.54%ID/g, respectively, which are comparable to that of [^68^Ga]Ga-AMBA (6.69 ± 1.03%ID/g). However, the pancreas uptake values of [^68^Ga]Ga-TacBOMB2 and [^68^Ga]Ga-TacBOMB3 were much lower than that of [^68^Ga]Ga-AMBA (1.30–2.41%ID/g vs 62.4%ID/g) (Figure 5 and Table S4). This suggests that the [Thz^14^]Bombesin(7–14) pharmacophore is a promising targeting vector for the design of GRPR-targeting radiopharmaceuticals with low pancreas uptake. Our data suggest that replacing the Leu^13^ψThz^14^ reduced peptide bond in [^68^Ga]Ga-TacsBOMB2 and [^68^Ga]Ga-TacsBOMB3 not only results in agonist ligands ([^68^Ga]Ga-TacBOMB2 and [^68^Ga]Ga-TacBOMB3) with preserved good GRPR binding affinity, but also their low pancreas uptake characteristics.

Compared with [^68^Ga]Ga-AMBA, [^68^Ga]Ga-TacBOMB2 also showed lower background uptake, resulting in better tumor-to-background contrast ratios (Figure 4 and Figure 6, and Table S4). This suggests that [^68^Ga]Ga-TacBOMB2 is a better imaging tracer than [^68^Ga]Ga-AMBA to detect GRPR-expressing malignant lesions. Interestingly, our previous study showed that [^68^Ga]Ga-TacsBOMB3 had significantly higher liver uptake (21.5 ± 5.04%ID/g) [28], while the liver uptake value of [^68^Ga]Ga-TacBOMB3 was only 0.65 ± 0.19%ID/g. This is most likely due to the increased hydrophilicity of [^68^Ga]Ga-TacBOMB3 (LogD_7.4_: −2.55 ± 0.03 for [^68^Ga]Ga-TacBOMB3 and −1.75 ± 0.04 for [^68^Ga]Ga-TacsBOMB3).

A blocking study was performed for [^68^Ga]Ga-TacBOMB2 on PC-3 tumor-bearing mice to confirm targeting specificity by co-injecting 100 μg of [D-Phe^6^,Leu-NHEt^13^,des-Met^14^]Bombesin(6–14). An 85% reduction in the average uptake of [^68^Ga]Ga-TacBOMB2 in PC-3 tumor xenografts was observed with the co-injection of [D-Phe^6^,Leu-NHEt^13^,des-Met^14^]Bombesin(6–14), confirming its specific uptake in tumors (Figure 7 and Table S4). Moreover, the average uptake values of [^68^Ga]Ga-TacBOMB2 in the pancreas and stomach were also reduced by 88% and 76%, respectively, indicating that there was specific uptake of [^68^Ga]Ga-TacBOMB2 in the pancreas and stomach as well, which is consistent with the reported physiological expression of GRPR in both organs [1,4,5].

In vivo stability studies revealed that [^68^Ga]Ga-AMBA was more stable than [^68^Ga]Ga-TacBOMB2 and [^68^Ga]Ga-TacBOMB3 in mouse plasma, as their intact fractions were 39.4 ± 10.8, 12.7 ± 2.93 and 27.3 ± 4.84%, respectively, at 15 min post-injection (Figures S1–S3). This indicates that the slightly higher tumor uptake of [^68^Ga]Ga-AMBA may also owe to its better in vivo stability other than its better binding affinity toward GRPR. When comparing [^68^Ga]Ga-TacBOMB2 with our previously reported [^68^Ga]Ga-TacsBOMB2, [^68^Ga]Ga-TacsBOMB2 was much more stable in vivo, with 83.3 ± 1.15% remaining intact at 15 min post-injection. This suggests that replacing the reduced peptide bond (Leu^13^ψThz^14^) with an amide bond results in potential cleavage site(s) for endogenous peptidases, leading to a reduction in stability. However, this also emphasizes that there is potential for improvement for [^68^Ga]Ga-TacBOMB2 and [^68^Ga]Ga-TacBOMB3 if their in vivo stability can be enhanced, likely by substituting some of the amino acids in the targeting sequences with their unnatural amino acids analogs.

In addition to RC-3950-II and RC-3965-II for the design of [^68^Ga]Ga-TacsBOMB2/[^68^Ga]Ga-TacBOMB2 and [^68^Ga]Ga-TacsBOMB3/[^68^Ga]Ga-TacBOMB3, respectively, there are other potent [AA^13^ψAA^14^]Bombesin-derived antagonists reported by the Schally group and others [23,24,34,35]. Our data suggest that these [AA^13^ψAA^14^]Bombesin-derived antagonists can be used directly for the design of GRPR-targeting antagonist radioligands, or alternatively, by replacing the (AA^13^ψAA^14^) reduced peptide bond with an amide bond for the design of GRPR-targeting agonist radioligands.

## 4. Materials and Methods

### 4.1. General Methods

AMBA and Ga-AMBA were synthesized following published procedures [36,37]. All the other chemicals and solvents were purchased from commercial sources and used without further purification. GRPR-targeting peptides were constructed on solid phase using an AAPPTec (Louisville, KY, USA) Endeavor 90 peptide synthesizer. The purification and quality control procedures for DOTA-conjugated peptides and their ^nat^Ga/^68^Ga-complexed analogs were performed on Agilent (Santa Clara, CA, USA) HPLC systems equipped with a model 1200 quaternary pump, a model 1200 UV absorbance detector (220 nm), and a Bioscan (Washington, DC, USA) NaI scintillation detector. The operation of Agilent HPLC systems was controlled using the Agilent ChemStation software version C.01.05. A semi-preparative column (Luna C18, 5 µm, 250 × 10 mm) and an analytical column (Luna C18, 5 µm, 250 × 4.6 mm), purchased from Phenomenex (Torrance, CA, USA), were used for purification and quality control. The HPLC eluates were collected and lyophilized with a Labconco (Kansas City, MO, USA) FreeZone 4.5 Plus freeze-drier. The MS analyses of DOTA-conjugated peptides and their ^nat^Ga-complexed analogs were conducted with a Waters (Milford, MA, USA) Acquity QDa mass spectrometer equipped with a 2489 UV/Vis detector and an e2695 Separations module. C18 Sep-Pak cartridges (1 cm^3^, 50 mg) were purchased from Waters (Milford, MA, USA). ^68^Ga was eluted from an ITM Medical Isotopes GmbH (Munich, Germany) generator, and purified according to the previously published procedures using a DGA resin column from Eichrom Technologies LLC (Lisle, IL, USA) [38]. The radioactivity of the ^68^Ga-labeled peptides was measured using a Capintec (Ramsey, NJ, USA) CRC^®^-25R/W dose calibrator, and the radioactivity of mouse tissues collected from biodistribution studies was counted using a Perkin Elmer (Waltham, MA, USA) Wizard2 2480 automatic gamma counter.

### 4.2. Synthesis of DOTA-Conjugated Peptides

TacBOMB2, TacBOMB3, and TacBOMB4 were synthesized on solid phase using Fmoc peptide chemistry. Rink Amide MBHA resin (0.05 mmol, 0.125 g) was treated with 20% piperidine in *N*,*N*-dimethylformamide (DMF) to remove the Fmoc-protecting group. Fmoc-protected amino acids (5 eq.), Fmoc-4-amino-(1-carboxymethyl)piperidine (5 eq.) were pre-activated with HATU (5 eq.), HOAt (5 eq.), and *N*,*N*-diisopropylethylamine (DIEA, 9 eq.) and then sequentially coupled to the resin. Then, DOTA(*t*Bu)_3_ (5 eq.), pre-activated with HATU (5 eq.) and DIEA (25 eq.), was coupled to the *N*-terminus of the peptides.

The peptides were deprotected and cleaved from the Rink Amide MBHA resin using a mixture of trifluoroacetic acid (TFA, 81.5%), triisopropylsilane (TIS 1.0%), water (5%), 2,2′-(ethylenedioxy)diethanethiol (DODT, 2.5%), thioanisole (5%), and phenol (5%) for 4 h at room temperature. After filtration, the cleaved peptides were precipitated by the addition of cold diethyl ether. The precipitated crude peptides were collected by centrifugation and then purified with HPLC (semi-preparative column; flow rate: 4.5 mL/min). The eluates containing the desired peptides were collected and lyophilized. The HPLC conditions, retention times, isolated yields, and MS confirmations of DOTA-conjugated peptides are provided in Table S1.

### 4.3. Synthesis of Nonradioactive Ga-Complexed Standards

The nonradioactive Ga-complexed standards were synthesized by incubating DOTA-conjugated precursor (1 eq.) and GaCl_3_ (5 eq.) in NaOAc buffer (0.1 M, 500 µL, pH 4.2–4.5) at 80 °C for 15 min. The reaction mixture was then purified via HPLC (semi-preparative column, flow rate: 4.5 mL/min). The HPLC eluates containing the desired peptide were collected and lyophilized. The HPLC conditions, retention times, isolated yields, and MS confirmations of the nonradioactive Ga-complexed standards are provided in Table S2.

### 4.4. Synthesis of ^68^Ga-Labeled Compounds

The radiolabeling experiments were performed following previously published procedures [38,39,40]. Purified ^68^GaCl_3_ in 0.5 mL of water was added to a 4 mL glass vial preloaded with 0.7 mL of HEPES buffer (2 M, pH 5.0) and 10 μL of precursor solution (1 mM). The radiolabeling reaction was carried out under microwave heating for 1 min before being purified by HPLC using the semi-preparative column. The eluate fraction containing the radiolabeled product was collected, diluted with water (50 mL), and passed through a C18 Sep-Pak cartridge that was pre-washed with ethanol (10 mL) and water (10 mL). After washing the C18 Sep-Pak cartridge with water (10 mL), the ^68^Ga-labeled product was eluted off the cartridge with ethanol (0.4 mL) containing 1% ascorbic acid and diluted with PBS containing 1% ascorbic acid for imaging and biodistribution studies. Quality control was performed using the analytical column. The HPLC conditions and retention times are provided in Table S3. The tracers were obtained in 51–80% decay-corrected radiochemical yields with 234 to 322 GBq/µmol molar activity and >95% radiochemical purity.

### 4.5. LogD_7.4_ Measurement

LogD_7.4_ values of [^68^Ga]Ga-TacBOMB2, [^68^Ga]Ga-TacBOMB3, and [^68^Ga]Ga-AMBA were measured using the shake flask method, as previously reported [38]. Briefly, aliquots (2 μL) of the ^68^Ga-labeled peptides were added into a 15 mL falcon tube containing 3 mL of n-octanol and 3 mL of 0.1 mol/L DPBS (pH 7.4). The mixture was vortexed for 1 min and then centrifuged at 3000 rpm for 15 min. Samples of the n-octanol (1 mL) and buffer (1 mL) layers were collected and measured in a gamma counter. LogD_7.4_ was calculated using the following equation: LogD_7.4_ = log_10_ [(counts in the n-octanol phase)/(counts in the buffer phase)].

### 4.6. Cell Culture

The PC-3 cells obtained from ATCC (via Cedarlane, Burlington, Canada) were cultured in RPMI 1640 medium (Life Technologies Corporations) supplemented with 10% FBS, penicillin (100 U/mL), and streptomycin (100 μg/mL) at 37 °C in a Panasonic Healthcare (Tokyo, Japan) MCO-19AIC humidified incubator containing 5% CO_2_. The cells were confirmed pathogen-free via an IMPACT Rodent Pathogen Test (IDEXX BioAnalytics). Cells grown to 80–90% confluence were washed with sterile DPBS (pH 7.4) and collected after 1 min of trypsinization. The cell concentration was counted in duplicate using a hemocytometer and a manual laboratory counter.

### 4.7. Fluorometric Calcium Release Assay

Following previously published procedures [41,42], 5 × 10^4^ PC-3 cells in 100 μL of growth media were seeded per well in a 96-well, clear bottom black plate 24 h before the assay. The loading buffer (100 μL/well), containing a calcium-sensitive dye (FLIPR Calcium 6 assay kit), was added to the 96-well plate. After incubation at 37 °C for 2 h, the plate was placed in a FlexStation 3 microplate reader (Molecular Devices). Ga-TacBOMB2 (50 nM), Ga-TacBOMB3 (50 nM), Ga-TacBOMB4 (50 nM), Ga-AMBA (50 nM), [D-Phe^6^,Leu-NHEt^13^,des-Met^14^]Bombesin(6–14) (50 nM, antagonist control), bombesin (50 nM, agonist control), adenosine triphosphate (ATP, 50 nM, positive control), or DPBS (blank control) was added to the cells, and the fluorescent signals were acquired for 2 min (λ_Ex_ = 485 nm; λ_Em_ = 525 nm; *n* = 2). Agonistic/antagonistic properties were determined using the relative fluorescent unit (RFU = max–min).

### 4.8. In Vitro Competition Binding Assay

PC-3 cells were seeded in 24-well, poly-D-lysine plates at 2 × 10^5^ cells/well 24–48 h prior to the assay. The growth medium was replaced with 400 μL of reaction medium (RPMI 1640 containing 2 mg/mL of BSA, and 20 mM of HEPES). Then, the plates were incubated for about 60 min at 37 °C. Ga-complexed nonradioactive standards of TacBOMB2, TacBOMB3, TacBOMB4, and AMBA in 50 μL of reaction medium with decreasing concentrations (10 μM to 1 pM) and 50 μL of 0.011 nM [^125^I-Tyr^4^]Bombesin were added into the wells, followed by incubation with moderate agitation for 1 h at 36 °C. Cells were gently washed with ice-cold PBS twice, harvested by trypsinization, and counted for radioactivity on a Perkin Elmer (Waltham, MA, USA) Wizard2 2480 automatic gamma counter. Data were analyzed using nonlinear regression (one binding site model for competition assay) with GraphPad (San Diego, CA, USA) Prism 8.4.3 software.

### 4.9. Ex Vivo Biodistribution, PET/CT Imaging and In Vivo Stability Studies

PET/CT imaging, biodistribution, and in vivo stability studies were conducted on male NOD.Cg-Rag1^tm1Mom^ Il2rg^tm1Wjl^/SzJ (NRG) mice, following previously published procedures [38,41,42,43]. The experiments were conducted according to the guidelines established by the Canadian Council on Animal Care and approved by the Animal Ethics Committee of the University of British Columbia (protocol number A20-0113, approved on 30 September 2020). The mice were anesthetized by inhalation of 2.5% isoflurane in 2 mL/min of oxygen and implanted subcutaneously with 5 × 10^6^ PC-3 cells (100 µL; 1:1 PBS/Matrigel) behind the left shoulder. Mice were used for PET/CT imaging and biodistribution studies when the tumor grew to 5–8 mm in diameter over around 4 weeks.

PET/CT imaging experiments were performed on a Siemens (Knoxville, TN) Inveon micro PET/CT scanner. The tumor-bearing mouse was injected with 3–5 MBq of ^68^Ga-labeled tracer through a lateral caudal tail vein under anesthesia, followed by recovery and roaming freely in its cage during the uptake period. At 50 min post-injection, a 10 min CT scan was conducted first for the localization and attenuation correction after segmentation for reconstructing the PET images, followed by a 10 min static PET imaging acquisition.

For biodistribution studies, the mice were injected with the radiotracer (2–4 MBq) via the tail vein as described above. For blocking, the mice were co-injected with 100 μg of [D-Phe^6^,Leu-NHEt^13^,des-Met^14^]Bombesin(6–14). At 1 h post-injection, the mice were anesthetized through isoflurane inhalation and euthanized through CO_2_ inhalation. Blood was collected through a cardiac puncture, and organs/tissues of interest were collected, weighed, and counted using a Perkin Elmer (Waltham, MA, USA) Wizard2 2480 automatic gamma counter.

For in vivo stability studies, 7–9 MBq of [^68^Ga]Ga-TacBOMB2, [^68^Ga]Ga-TacBOMB3, or [^68^Ga]Ga -AMBA was injected via the lateral caudal vein into healthy male NRG mice (*n* = 3). At 15 min post-injection, mice were sedated and euthanized, and urine and blood were collected. The plasma was extracted from whole blood by adding CH_3_CN (500 μL), vortex, centrifugation, and the separation of supernatant. The plasma and urine samples were analyzed via radio-HPLC using the conditions for quality control of these ^68^Ga-labeled radioligands (Table S3).

### 4.10. Statistical Analysis

Statistical analyses were performed using Student’s *t*-test in Microsoft (Redmond, WA, USA) Excel 2007 software. The comparison of biodistribution data between [^68^Ga]Ga-TacBOMB2 and [^68^Ga]Ga-AMBA was conducted via an unpaired, two-tailed test. An unpaired, one-tailed test was used to compare the biodistribution data of [^68^Ga]Ga-TacBOMB2 with/without the co-injection of [D-Phe^6^,Leu-NHEt^13^,des-Met^14^]Bombesin(6–14). A statistically significant difference was considered when the adjusted *p*-value was <0.05.

## 5. Conclusions

Replacing the (Leu^13^ψThz^14^) reduced peptide bond in the previously reported GRPR antagonist tracers, [^68^Ga]Ga-TacsBOMB2 and [^68^Ga]Ga-TacsBOMB3, retains their high GRPR binding affinity, but the resulting [^68^Ga]Ga-TacBOMB2 and [^68^Ga]Ga-TacBOMB3 become GRPR agonists. Similar to [^68^Ga]Ga-TacsBOMB2 and [^68^Ga]Ga-TacsBOMB3, the derived [^68^Ga]Ga-TacBOMB2 and [^68^Ga]Ga-TacBOMB3 agonist PET tracers also retain in vivo GRPR-targeting capabilities, as demonstrated by their good tumor uptake and tumor-to-background contrast ratios in imaging and biodistribution studies. Compared with the clinically validated agonist PET tracer [^68^Ga]Ga-AMBA, [^68^Ga]Ga-TacBOMB2 has comparable tumor uptake but higher tumor-to-background contrast ratios. Therefore, [^68^Ga]Ga-TacBOMB2 is promising for clinical development to detect GRPR-expressing tumors with PET. Due to the agonist characteristics, potentially longer tumor retention, and negligible pancreatic uptake of [^68^Ga]Ga-TacBOMB2 and [^68^Ga]Ga-TacBOMB3, [Thz^14^]Bombesin(7–14) is a promising vector for the design of GRPR-targeting radiopharmaceuticals, particularly for radioligand therapy applications to minimize toxicity to the pancreas.

## 6. Patents

The compounds disclosed in this report are covered by a recent US provisional patent application (Serial number 63/323,831; filing date: 25 March 2022). Lei Wang, Zhengxing Zhang, Ivica Jerolim Bratanovic, François Bénard, and Kuo-Shyan Lin are listed as inventors of this filed patent.

## Data Availability

The data presented in this study are available in the Supplementary Materials.

## Supplementary Materials

The following supporting information can be downloaded at: https://www.mdpi.com/article/10.3390/molecules28041977/s1, Table S1: HPLC purification conditions and MS characterizations of TacBOMB2, TacBOMB3, and TacBOMB4; Table S2: HPLC purification conditions and MS characterizations of Ga-TacBOMB2, Ga-TacBOMB3, and Ga-TacBOMB4; Table S3: HPLC conditions for the purification and quality control of ^68^Ga-labeled TacBOMB2, TacBOMB3, and AMBA; Table S4: Biodistribution and uptake ratios of ^68^Ga-labeled GRPR-targeting tracers in PC-3 tumor-bearing mice; Figure S1: Representative radio-HPLC chromatograms from analysis of intact fraction of [^68^Ga]Ga-TacBOMB2 in mouse plasma and urine samples collected at 15 min post-injection; Figure S2: Representative radio-HPLC chromatograms from analysis of intact fraction of [^68^Ga]Ga-TacBOMB3 in mouse plasma and urine samples collected at 15 min post-injection; Figure S3: Representative radio-HPLC chromatograms from analysis of intact fraction of [^68^Ga]Ga-AMBA in mouse plasma and urine samples collected at 15 min post-injection.

## Sample Availability

Not available.
