# Supplementary material for: 68Ga-Labeled [Thz14]Bombesin(7–14) Analogs: Promising GRPR-Targeting Agonist PET Tracers with Low Pancreas Uptake
Source: Molecules. 2023 Feb 20;28(4):1977. doi: 10.3390/molecules28041977 (PMC9962964; doi:10.3390/molecules28041977)
Supplement: Supplementary file 1 [file molecules-28-01977-s001.zip › molecules-2231886-supplementary.pdf]

SUPPLEMENTAL INFORMATION

Table S1: HPLC purification conditions and MS characterizations of TacBOMB2, TacBOMB3, and TacBOMB4.

Compound name	HPLC conditions	Retention time (min)	Yield (%)	Calculated mass (m/z)	Found (m/z)
TacBOMB2	23% CH_3CN and 0.1% TFA in H_2O	11.3	30	$[M+2H]^{2+}$ 799.4	$[M+2H]^{2+}$ 799.6
TacBOMB3	25% CH_3CN and 0.1% TFA in H_2O	13.0	38	$[M+2H]^{2+}$ 824.4	$[M+2H]^{2+}$ 824.9
TacBOMB4	25% CH_3CN and 0.1% TFA in H_2O	15.9	55	$[M+2H]^{2+}$ 824.9	$[M+2H]^{2+}$ 825.1

Table S2: HPLC purification conditions and MS characterizations of Ga-TacBOMB2, Ga-TacBOMB3, and Ga-TacBOMB4.

Compound name	HPLC conditions	Retention time (min)	Yield (%)	Calculated mass (m/z)	Found (m/z)
Ga-TacBOMB2	23% CH_3CN and 0.1% TFA in H_2O	12.0	82	$[M+2H]^{2+}$ 833.9	$[M+2H]^{2+}$ 833.7
Ga-TacBOMB3	25% CH_3CN and 0.1% TFA in H_2O	13.9	63	$[M+2H]^{2+}$ 858.9	$[M+2H]^{2+}$ 858.5
Ga-TacBOMB4	25% CH_3CN and 0.1% TFA in H_2O	17.3	58	$[M+2H]^{2+}$ 859.4	$[M+2H]^{2+}$ 858.8

Table S3: HPLC conditions for the purification and quality control of ^{68}Ga-labeled TacBOMB2 TacBOMB3, and AMBA. FA: formic acid.

Compound name	HPLC conditions		Retention time (min)
[^{68}Ga]Ga-TacBOMB2	Semi-Prep	19% CH_3CN and 0.1% FA in H_2O; flow rate 4.5 mL/min	12.1
	QC	21% CH_3CN and 0.1% FA in H_2O; flow rate 2.0 mL/min	5.8
[^{68}Ga]Ga-TacBOMB3	Semi-Prep	19% CH_3CN and 0.1% FA in H_2O; flow rate 4.5 mL/min	17.0
	QC	22% CH_3CN and 0.1% FA in H_2O; flow rate 2 mL/min	4.7
[^{68}Ga]Ga-AMBA	Semi-Prep	19% CH_3CN and 0.1% FA in H_2O; flow rate 4.5 mL/min	24.8
	QC	18.5% CH_3CN and 0.1% FA in H_2O; flow rate 2.0 mL/min	6.5

Table S4: Biodistribution (mean ± SD, n = 4) and uptake ratios of ^{68}Ga-labeled GRPR-targeting tracers in PC-3 tumor-bearing mice. The mice in the blocked group were co-injected with 100 µg of [D-Phe^{6},Leu-NHEt^{13},des-Met^{14}]Bombesin(6-14). * and ** indicate p < 0.05 and < 0.01, respectively, when comparing the 1 h and 1 h blocked data of [^{68}Ga]Ga-TacBOMB2.

Tissue (%ID/g)	[^{68}Ga]Ga-TacBOMB2		[^{68}Ga]Ga-TacBOMB3	[^{68}Ga]Ga-AMBA
	1 h	1 h blocked	1 h	1 h
Blood	0.39 ± 0.12	0.55 ± 0.19	1.06 ± 0.51	0.58 ± 0.07
Fat	0.10 ± 0.03	0.07 ± 0.02	0.19 ± 0.18	0.08 ± 0.02
Testes	0.16 ± 0.05	0.21 ± 0.16	0.37 ± 0.06	0.20 ± 0.03
Intestines	0.60 ± 0.12	0.28 ± 0.07**	1.06 ± 0.26	8.62 ± 1.00
Spleen	0.27 ± 0.10	0.18 ± 0.05	0.50 ± 0.32	2.49 ± 2.16
Pancreas	1.30 ± 0.14	0.15 ± 0.03**	2.41 ± 0.72	62.4 ± 4.26
Stomach	0.54 ± 0.27	0.13 ± 0.06*	0.82 ± 0.37	2.32 ± 0.60
Liver	0.29 ± 0.13	0.29 ± 0.06	0.65 ± 0.19	0.43 ± 0.05
Kidneys	2.43 ± 0.16	1.99 ± 0.59	4.61 ± 2.72	5.70 ± 2.45
Heart	0.14 ± 0.02	0.16 ± 0.04	0.35 ± 0.18	0.22 ± 0.02
Lungs	0.39 ± 0.10	0.39 ± 0.10	1.63 ± 0.75	0.62 ± 0.06
PC-3 tumor	5.95 ± 0.50	0.92 ± 0.22**	5.09 ± 0.54	6.69 ± 1.03
Bone	0.26 ± 0.11	0.10 ± 0.03*	0.19 ± 0.09	0.33 ± 0.13
Muscle	0.13 ± 0.08	0.13 ± 0.04	0.24 ± 0.12	0.17 ± 0.01
Brain	0.03 ± 0.01	0.02 ± 0.00	0.04 ± 0.02	0.04 ± 0.01
Tumor/bone	28.9 ± 18.3	8.94 ± 1.49*	29.9 ± 8.50	22.6 ± 9.13
Tumor/muscle	57.9 ± 32.8	7.14 ± 1.40*	23.5 ± 9.61	39.5 ± 7.46
Tumor/blood	16.4 ± 5.64	1.71 ± 0.18**	5.61 ± 2.33	11.7 ± 2.10
Tumor/intestines	10.5 ± 3.06	3.33 ± 0.67**	4.93 ± 0.84	0.79 ± 0.22
Tumor/kidney	2.46 ± 0.33	0.47 ± 0.04**	1.34 ± 0.58	1.36 ± 0.73
Tumor/pancreas	4.64 ± 0.77	6.22 ± 0.67*	2.21 ± 0.40	0.11 ± 0.01

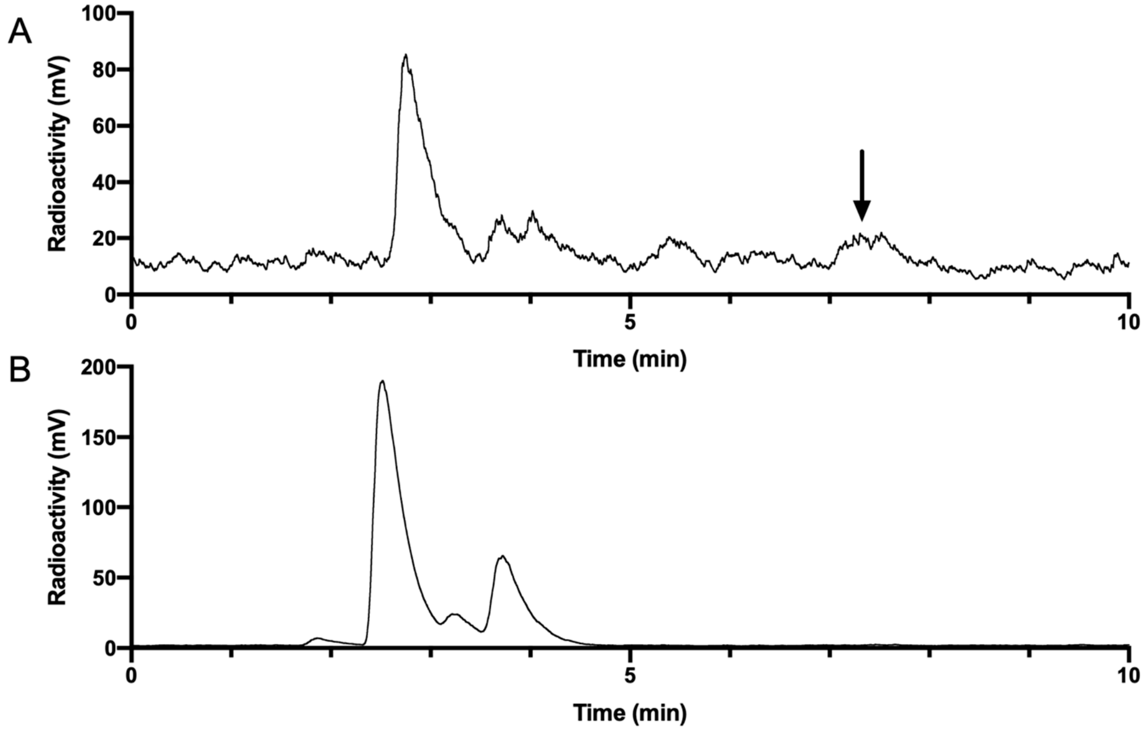

Figure S1. Representative radio-HPLC chromatograms from analysis of intact fraction of [^{68}Ga]Ga-TacBOMB2 in mouse plasma (A) and urine (B) samples collected at 15 min post-injection. The peak pointed by an arrow is the intact tracer.

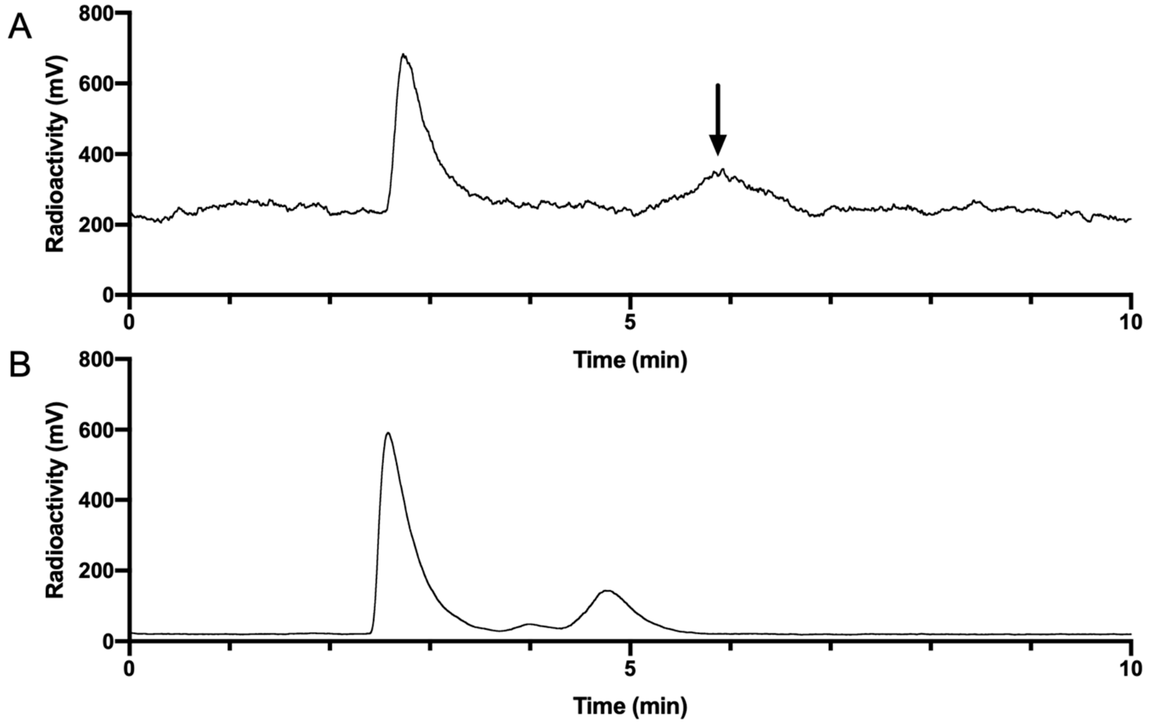

Figure S2. Representative radio-HPLC chromatograms from analysis of intact fraction of [^{68}Ga]Ga-TacBOMB3 in mouse plasma (A) and urine (B) samples collected at 15 min post-injection. The peak pointed by an arrow is the intact tracer.

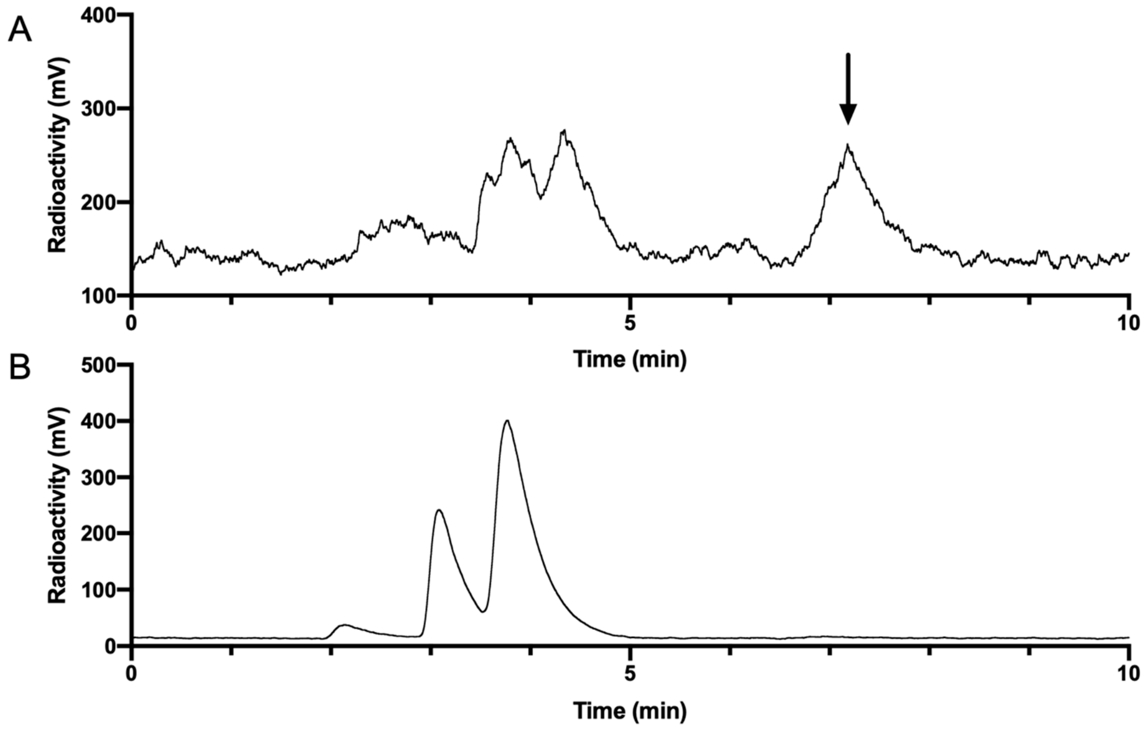

Figure S3. Representative radio-HPLC chromatograms from analysis of intact fraction of [^{68}Ga]Ga-AMBA in mouse plasma (A) and urine (B) samples collected at 15 min post-injection. The peak pointed by an arrow is the intact tracer.
